# Budding and Fission of Nanovesicles Induced by Membrane Adsorption of Small Solutes

**DOI:** 10.1021/acsnano.1c00525

**Published:** 2021-04-05

**Authors:** Rikhia Ghosh, Vahid Satarifard, Andrea Grafmüller, Reinhard Lipowsky

**Affiliations:** Theory & Biosystems, Max Planck Institute of Colloids and Interfaces, 14424 Potsdam, Germany

**Keywords:** lipid bilayer, nanovesicle, solute adsorption, vesicle budding, membrane neck, neck fission, vesicle division

## Abstract

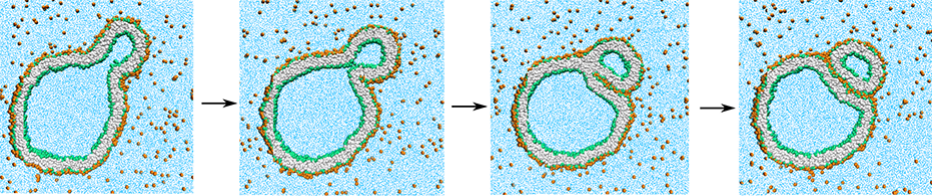

Membrane budding and fission are essential cellular processes that produce new membrane compartments during cell and organelle division, for intracellular vesicle trafficking as well as during endo- and exocytosis. Such morphological transformations have also been observed for giant lipid vesicles with a size of many micrometers. Here, we report budding and fission processes of lipid nanovesicles with a size below 50 nm. We use coarse-grained molecular dynamics simulations, by which we can visualize the morphological transformations of individual vesicles. The budding and fission processes are induced by low concentrations of small solutes that absorb onto the outer leaflets of the vesicle membranes. In addition to the solute concentration, we identify the solvent conditions as a second key parameter for these processes. For *good* solvent conditions, the budding of a nanovesicle can be controlled by reducing the vesicle volume for constant solute concentration or by increasing the solute concentration for constant vesicle volume. After the budding process is completed, the budded vesicle consists of two membrane subcompartments which are connected by a closed membrane neck. The budding process is reversible as we demonstrate explicitly by reopening the closed neck. For *poor* solvent conditions, on the other hand, we observe two unexpected morphological transformations of nanovesicles. Close to the binodal line, at which the aqueous solution undergoes phase separation, the vesicle exhibits recurrent shape changes with closed and open membrane necks, reminiscent of flickering fusion pores (kiss-and-run) as observed for synaptic vesicles. As we approach the binodal line even closer, the recurrent shape changes are truncated by the fission of the membrane neck which leads to the division of the nanovesicle into two daughter vesicles. In this way, our simulations reveal a nanoscale mechanism for the budding and fission of nanovesicles, a mechanism that arises from the interplay between membrane elasticity and solute-mediated membrane adhesion.

## Introduction

Biomembranes exhibit a fascinating variety of different morphologies and transformations between these morphologies. These morphological transformations are essential for important biological processes such as cell and organelle division, intracellular vesicle trafficking, as well as endo- and exocytosis.^1,2^ The corresponding remodeling processes involve membrane budding as an intermediate step. During budding, the membrane forms two subcompartments that are connected by a closed membrane neck. Subsequently, the neck is often cleaved by membrane fission which leads to the division of the cell or organelle and to the formation of two separate membrane compartments. Such budding and fission processes have also been observed for biomimetic model systems as provided by giant unilamellar vesicles (GUVs).^3−6^

GUVs have a typical diameter of many μm which implies that their morphological responses can be directly observed in the optical microscope. Here, we consider the polymorphism and shape transformations of nanovesicles with a much smaller diameter, in the range of 20 to 200 nm. Such nanovesicles can be produced from lipid dispersions by a variety of preparation methods such as extrusion through the pores of rigid filters^7,8^ and microfluidic mixing.^9^ Nanovesicles are also abundant *in vivo*. Examples are provided by synaptic vesicles, which have a diameter that varies between 20 and 50 nm,^10,11^ and by exosomes, small extracellular vesicles with a diameter between 25 and 100 nm.^12−14^ These exosomes are increasingly investigated as biomarkers for diseases and as targeted drug delivery systems.^15−18^

Nanovesicles with a diameter below 300 nm cannot be imaged by conventional optical microscopy. Therefore, a variety of electron microscopy (EM) methods has been used to obtain images of nanovesicles, but all EM methods are restricted to a single snapshot of each nanovesicle and, thus, cannot monitor how the morphology of such a vesicle changes with time. These morphological transformations are, however, accessible to molecular simulations as we have demonstrated in a recent study.^19^ In this previous simulation study, we showed that the morphologies and morphological transformations of a nanovesicle depend on the numbers of lipids which are initially assembled into the outer and inner leaflets of the bilayer membranes. Therefore, by varying this transbilayer distribution of the lipids, we could directly control the shape transformations that the nanovesicles underwent when we reduced the vesicle volume. The transbilayer lipid distribution is, however, difficult to use as an experimental control parameter. In order to facilitate the comparison between simulations and experimental studies, we introduce here another control parameter which is directly accessible to experiment. This parameter is provided by the concentration of small solutes that adsorb onto the outer leaflet of the nanovesicles. Examples for such solutes are metal ions which adsorb onto both charged^20−22^ and neutral^23,24^ membranes, small sugar molecules,^4,25−28^ and short PEG chains.^29−31^

**Figure 1:**
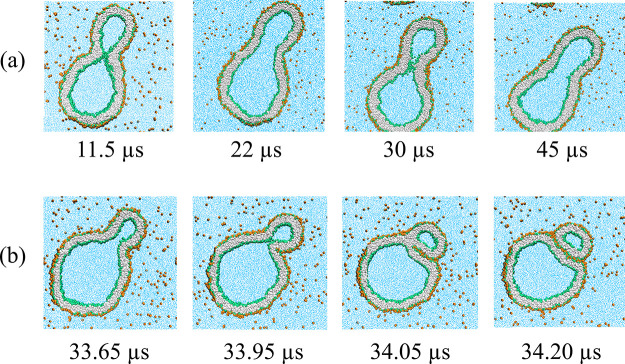
Unusual morphological transitions of nanovesicles exposed to small solutes (orange dots) in the exterior aqueous solution (blue) for low solute concentrations Φ_S_ and poor solvent conditions. The cross sections of the vesicle membranes depict lipid bilayers with green head groups and gray hydrocarbon chains. The solutes form adsorption layers (orange-green) at the outer leaflets of the bilayers: (a) Time series of budded nanovesicle with recurrent shape changes between dumbbells with open and closed necks for solute mole fraction Φ_S_ = 0.025. The membrane neck of the dumbbell is closed for time *t* = 11.5 μs, open for *t* = 22 μs, again closed for *t* = 30 μs, and again open for *t* = 45 μs, see also time-lapse Movie 1 and more detailed time series in Figures 6 and 7. (b) Division of nanovesicle by fission of membrane neck for Φ_S_ = 0.026. The membrane neck is open at *t* = 33.65 μs and closed at *t* = 33.95 μs, after which the membrane forms a rapidly growing contact area; see snapshot for *t* = 34.05 μs. The solute-mediated membrane adhesion leads to fission of the neck and division of the nanovesicle into two daughter vesicles for *t* ≥ 34.15 μs. For more details, see time-lapse Movie 2 and Figure 9.

In addition to the solute concentration, the solvent conditions are shown to represent a second key parameter, which determines the morphological responses of the nanovesicles. For good solvent conditions, the aqueous solution remains in a uniform liquid phase for all solute concentrations. Increasing the solute concentration then leads to the budding of the nanovesicle and to the formation of a closed membrane neck. This morphological transformation is reversible as we demonstrate by decreasing the solute concentration again. For poor solvent conditions, on the other hand, the aqueous solution undergoes phase separation beyond a certain concentration threshold corresponding to the binodal line. Close to the binodal line, the nanovesicle exhibits unusual morphological responses corresponding to recurrent changes between dumbbell shapes with open and closed necks, see Figure 1a, as well as fission of the membrane neck and division of the nanovesicle into two daughter vesicles as in Figure 1b. Here and below, the solute concentration is expressed in terms of the solute mole fraction Φ_S_ of the aqueous solution. The nanovesicle in Figure 1 undergoes recurrent shape changes for solute concentration Φ_S_ = 0.025 as well as neck fission for the slightly increased value Φ_S_ = 0.026. Thus, close to the binodal line, the behavior of the nanovesicle is found to be very sensitive to small changes in the solute concentration.

A variety of aqueous solutions undergo phase separation into two liquid phases. A classic example for such aqueous two-phase or biphasic systems is provided by aqueous solutions of polyethylene glycol (PEG) with low molecular weight and water-soluble salts such as tripotassium phosphate.^32,33^ Other two-phase systems are obtained from aqueous solutions of sodium thiosulfate and a variety of alcohols.^34,35^ For all of these aqueous solutions, the binodal of the liquid–liquid coexistence region has been determined experimentally. In order to reduce the number of model parameters, we will combine two components of such a ternary mixture into a single one and consider a binary instead of a ternary liquid mixture.

## Results and Discussion

### Key Parameters for the Assembly of Nanovesicles

Using the molecular model and computational approach described in the Methods, we first assemble spherical nanovesicles in plenty of water. We use coarse-grained lipid and water molecules which are built up from beads with a diameter *d* of about 0.8 nm. All nanovesicles discussed here were assembled from *N*_il_ = 4000 lipids in the inner leaflet and *N*_ol_ = 6100 lipids in the outer leaflet. The vesicle membrane divides the simulation box into two aqueous compartments. The interior compartment enclosed by the inner leaflet consists of *N*_W_^in^ water beads and no solute, while the exterior compartment in contact with the outer leaflet represents an aqueous solution of *N*_W_^ex^ water beads and *N*_S_ solute beads.

The initial volume of each spherical nanovesicle is set by the initial number *N*_W_^in^ = *N*_W_^isp^ of water beads enclosed by the nanovesicle. As a reference volume, we use *N*_W_^isp^ = 90 400 water beads, corresponding to a vesicle diameter of 45 *d* or 36 nm. We then study the shape transformations of these nanovesicles in response (i) to changes in the vesicle volume and (ii) to the adsorption of small solutes from the exterior aqueous solution.

Changes in the vesicle volume will be controlled by the number *N*_W_^in^ of interior water beads. To monitor these volume changes, we use the dimensionless volume parameter^19^
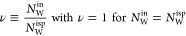
1

By decreasing and increasing this parameter, we mimic the experimental procedures of osmotic deflation and inflation. The solute adsorption from the exterior solution is controlled by the solute concentration which we measure in terms of the solute mole fraction
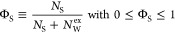
2where *N*_S_ is the number of solute beads and *N*_W_^ex^ is the number of water beads in the exterior solution. The limiting case of pure water without solute, corresponding to Φ_S_ = 0, was previously studied in ref (19). For a finite solute concentration, the solutes form an adsorption layer onto the outer leaflet of the lipid bilayers, thereby modulating the compositional asymmetry between the two leaflets.

The vesicle volume ν and the solute mole fraction Φ_S_ represent two key parameters for the morphologies of the nanovesicles. In addition, we will also show that these morphologies are strongly affected by the solubility ζ of the solutes in water, as defined in terms of the force parameters by eq 7 in the Methods. The solubility ζ and the solute mole fraction Φ_S_ determine the phase diagram of the aqueous solution as displayed in Figure S1 of the Supporting Information. The computational method to determine this phase diagram is illustrated in Figures S2 and S3.

For good solvent conditions, the solution remains uniform over the whole range of solute concentrations corresponding to mole fractions Φ_S_ with 0 ≤ Φ_S_ ≤ 1. In contrast, for poor solvent conditions, the solution undergoes phase separation into two coexisting liquid phases for intermediate solute concentrations. Therefore, as we increase the solute concentration for poor solvent conditions, the solution remains uniform only until we reach the binodal line, which separates the uniform one-phase region from the two-phase coexistence region of the phase diagram (Figure S1). To illustrate the different behavior for the two solvent conditions, we will study and compare two values of the solubility, ζ = 25/32 for good solvent and ζ = 25/40 for poor solvent conditions, corresponding to the blue and red horizontal lines in Figure S1. For poor solubility with ζ = 25/40, the binodal is already reached for solute concentration Φ_S_ = 0.0275 (Figures S2 and S3).

### Nanovesicles Exposed to Very Dilute Solutions

As a first simple example, let us consider nanovesicles that are exposed to very dilute solutions with solute concentration Φ_S_ = 0.016. In this case, the morphological response of the nanovesicle to changes of its volume is essentially independent of the solvent conditions. The latter behavior is illustrated in Figures S4 and S5 for good and poor solvent conditions, respectively. For both conditions, the nanovesicle undergoes essentially the same series of shape transformations as we reduce the vesicle volume ν by reducing the number *N*_W_^in^ of water beads within the vesicle. In fact, this series of shape transformations is very similar to the one previously observed^19^ in the absence of solutes.

### Vesicle Budding and Neck Closure for Good Solvent Conditions

**Figure 2:**
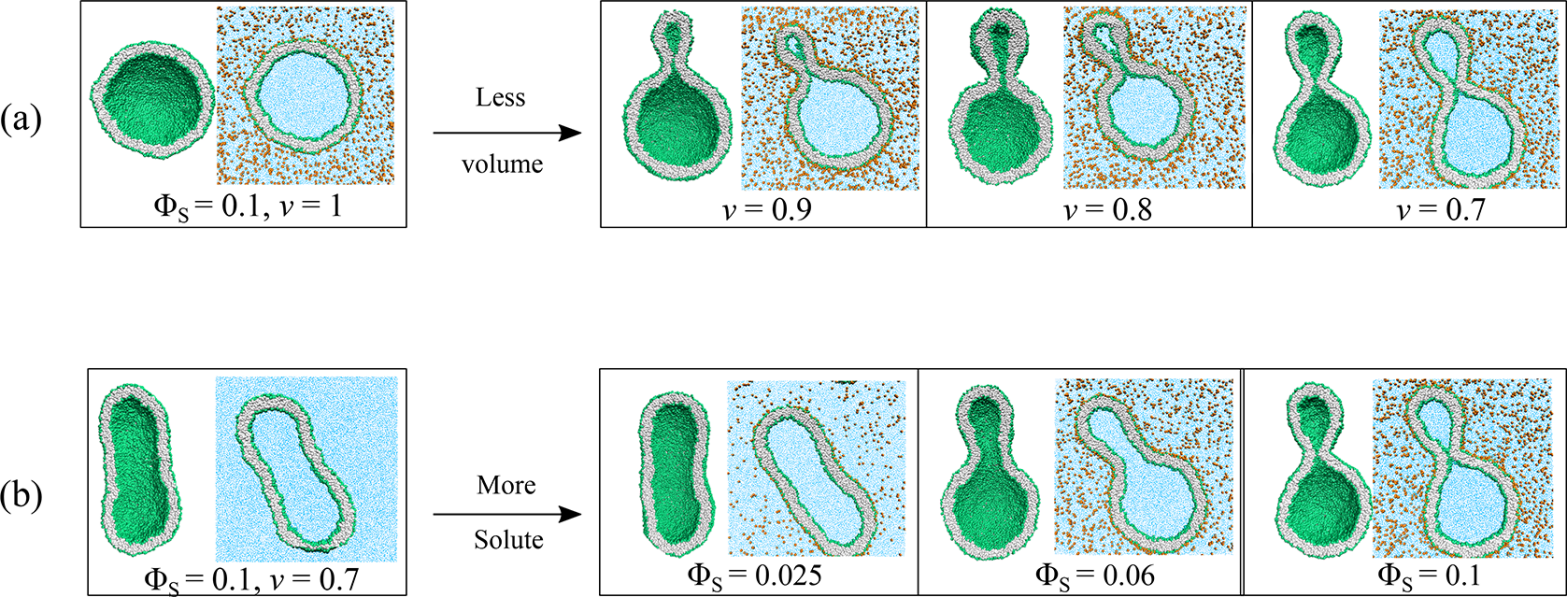
Budding of nanovesicles for good solvent conditions. (a) A spherical vesicle with volume parameter ν = 1 is exposed to solute mole fraction Φ_S_ = 0.1 and subsequently deflated until ν = 0.7. (b) The same spherical vesicle is first deflated to ν = 0.7 and then exposed to an increasing solute mole fraction up to Φ_S_ = 0.1. Both protocols lead to the same final dumbbell morphology with a closed neck, corresponding to the rightmost snapshots.

Budded shapes of nanovesicles with closed membrane necks can be generated from spherical vesicle shapes using two different protocols based on changing the vesicle volume or the solute concentration in the exterior solution. First, we can generate budded shapes with closed membrane necks by exposing a spherical nanovesicle to a sufficiently large solute concentration and then reducing the volume of this vesicle; see Figure 2a. Second, the same budded shapes can also be formed when we first deflate the vesicle in the absence of solute and subsequently increase the solute concentration in the exterior solution; see Figure 2b. For a deflated vesicle with volume parameter *v* = 0.7, the membrane neck closes when we reach a threshold value of the solute concentration Φ_S_ that is larger than 0.09 and smaller than 0.1. The latter budding process is reversible as we demonstrate by decreasing and increasing the solute concentration several times; see the snapshots in Figure S6.

### Density of Adsorbed Solutes and Solute Coverage

The shape transformations displayed in Figures 2b and S6 are caused by changing the solute concentration and thus the solute coverage, i.e., the amount of solute adsorbed onto the outer leaflet of the nanovesicle. The dependence of the solute coverage on the solute concentration can be computed in a quantitative manner if we consider spherical vesicles, for which the solute density ρ_S_ depends only on the radial coordinate *r*.

**Figure 3:**
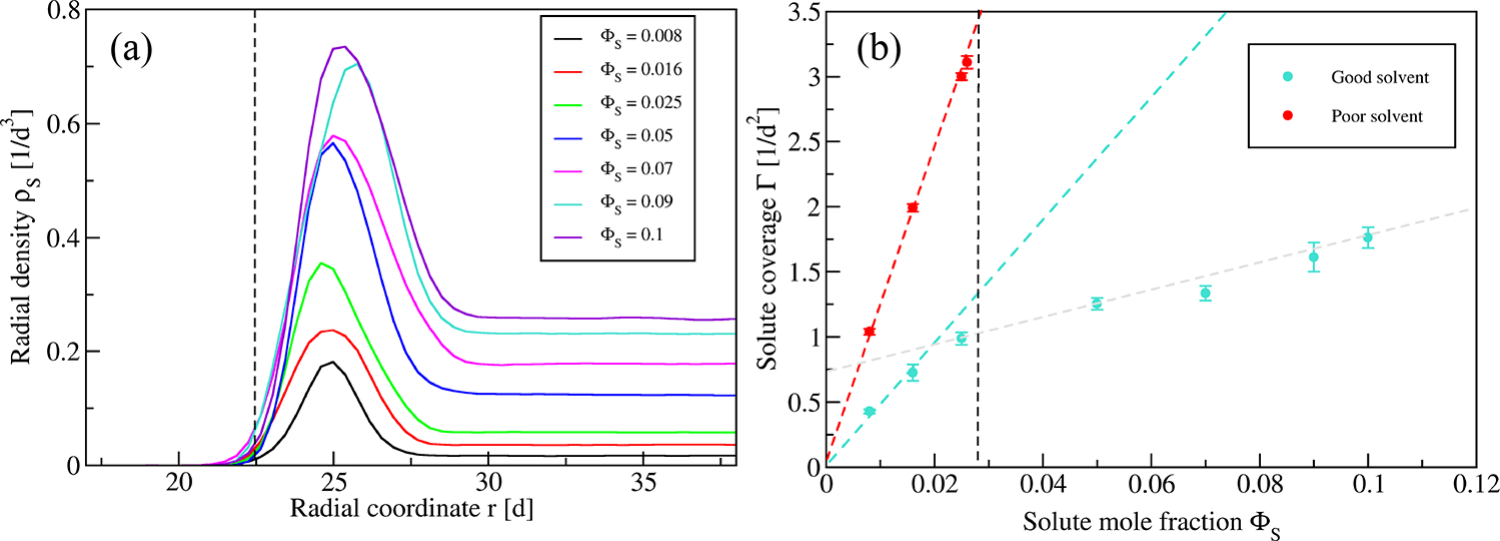
Spherical nanovesicles with tensionless bilayers exposed to different solute concentrations Φ_S_. (a) Solute density profiles ρ_S_ as a function of the radial coordinate *r* for good solvent conditions. For each mole fraction, the profile attains a constant value for *r* ≳ 28*d*. The vertical dotted line at *r* = 22.5*d* corresponds to the average value of the midsurface radius *R*_mid_, see Table S1. (b) Monotonic increase of solute coverage Γ of the outer leaflet with solute concentration Φ_S_, both for good solvent (cyan data) and for poor solvent (red data) conditions. The vertical dashed line at Φ_S_ = 0.0275 corresponds to the location of the binodal, at which the aqueous solution starts to undergo phase separation for poor solubility ζ = 25/40. For both solvent conditions, the coverage Γ is computed by eq 4; for poor solvent conditions, Γ is found to increase rather strongly.

In Figure 3a, we display the solute density profiles ρ_S_(*r*) for tensionless bilayers at several solute concentrations. For each concentration, the solute density profile attains a constant value for *r* ≳ 28*d* which represents the bulk value ρ_S,b_ of the solute density. The corresponding excess profiles
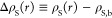
3decay rapidly to zero as we move away from the outer bilayer leaflet toward larger *r* values. The solute coverage Γ of the outer leaflet is then obtained by integrating this excess profile over the volume *V*^ex^ of the exterior solution.

To specify this exterior volume, we take the vesicle radius to be the radius of the bilayer’s midsurface *R*_mid_, at which the hydrophobic lipid chains exhibit a pronounced density maximum, see Methods. The resulting numerical values of *R*_mid_ are included in Tables S1 and S2 for good and poor solvent conditions. Inspection of the solute density profiles in Figure 3a shows that the interior vesicle volume with *r* < *R*_mid_ is essentially free of solutes for all solute concentrations. The surface area of the vesicle is then taken to be *A*_0_ = 4 *π R*_mid_^2^. As a consequence, we obtain the coverage
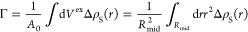
4of the outer leaflet which increases with the solute concentration Φ_S_ as shown in Figure 3b for both solvent conditions. The numerical Γ values are provided in the sixth columns of Tables S1 and S2 for good and poor solvent conditions, respectively.

Comparing the values for the two solvent conditions, we conclude that the coverage Γ increases more strongly for poor solvent conditions. A simple estimate for this enhanced increase of the coverage can be obtained from the straight fitting lines that have been included in Figure 3b for small Φ_*S*_. These fitting lines have the functional form Γ = *b*_Γ_ Φ_S_/*d*^2^ with the dimensionless coefficient *b*_Γ_ = 45.6 for good and *b*_Γ_ = 124.4 for poor solubility. Therefore, for the same solute concentrations, the coverage Γ is about 2.7 times larger for poor solvent conditions with solubility ζ = 25/40 than for good solvent conditions with solubility ζ = 25/32.

The solute coverage could also be estimated by considering local contacts between the solute (S) beads and the headgroup (H) beads of the lipids. However, in order to define SH contacts, we would have to introduce a certain molecular length scale *l*_SH_ and take the beads to be in contact if their separation is smaller than *l*_SH_. This length scale should be of the order of the bead diameter *d* but its precise magnitude is somewhat arbitrary. This arbitrariness is avoided by computing the coverage from the excess profile Δρ_S_(*r*) of the solute density as in eq 4.

### Adsorption-Induced Stress Asymmetry and Spontaneous Curvature

**Figure 4:**
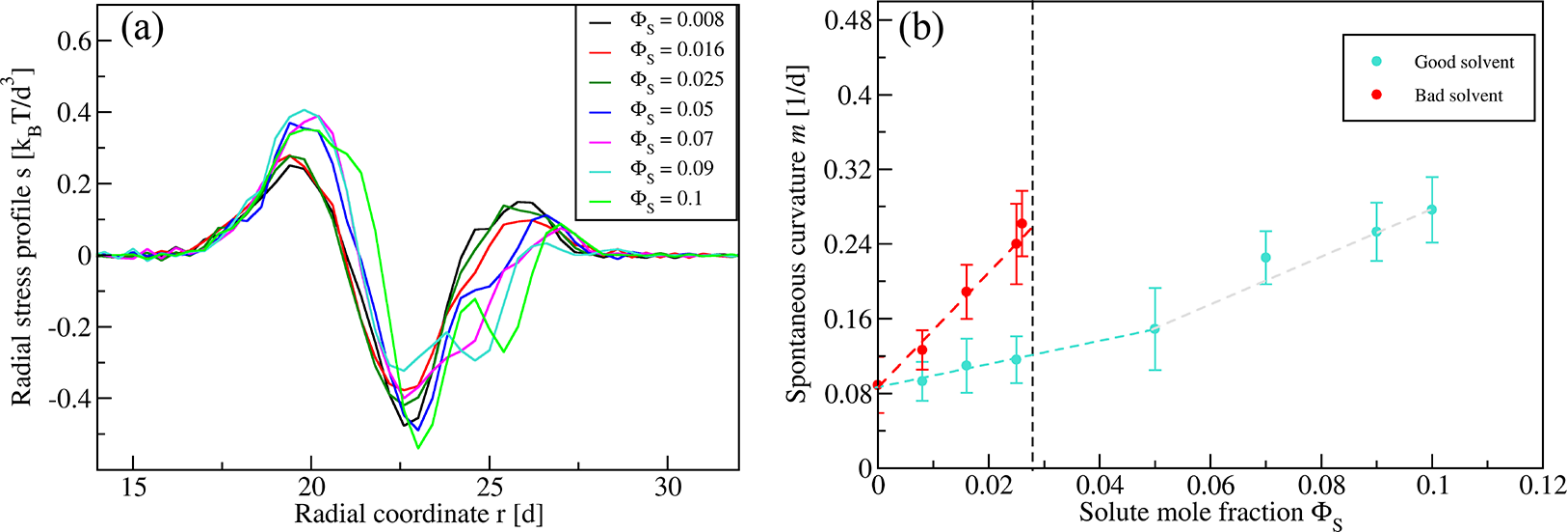
Stress profiles and spontaneous curvature for spherical vesicles exposed to different solute concentrations. (a) Stress profiles *s*(*r*) for good solvent conditions and seven different values of the solute concentration Φ_S_, see inset. (b) Monotonic increase of spontaneous curvature *m* with solute concentration Φ_S_, both for good solvent conditions (cyan data) and for poor solvent conditions (red data). The vertical dashed line at Φ_S_ = 0.0275 corresponds to the location of the binodal for poor solubility ζ = 25/40. For both solvent conditions, the spontaneous curvature is calculated by eq 5; for poor solvent conditions, this curvature increases rather strongly, in particular close to the binodal line at Φ_S_ = 0.0275. Comparison of the data in (b) with those in Figure 3b shows that the functional dependence of the spontaneous curvature and the solute coverage on the solute concentration are quite similar.

The adsorption of an increasing amount of solute onto the outer bilayer leaflet leads to an increasing bilayer asymmetry which implies an increasing stress asymmetry or spontaneous curvature as obtained from the first moment of the stress profile. As explained in the Methods, see eqs 8 and 9, the stress profile of a spherical vesicle depends only on the radial coordinate *r*. Examples for such stress profiles, *s*(*r*), are displayed in Figure 4a for seven different solute concentrations. Using these stress profiles, the spontaneous curvature *m* is calculated *via* the relationship^19^
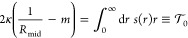
5between the bending rigidity κ, the midsurface radius *R*_mid_ of the vesicle, the spontaneous curvature *m*, and the first moment  of the stress profile *s*(*r*), where the subscript 0 of  indicates vanishing bilayer tension. The resulting values of the first moment  and the spontaneous curvature *m* are displayed in the last columns of Tables S1 and S2 for good and poor solvent conditions, respectively. These tables also contain the bending rigidity κ, which enters eq 5 for the spontaneous curvature, and the area compressibility modulus *K*_*A*_, which was used to compute the bending rigidity κ using the relationship^36^ κ = *K*_A_*l*_me_^2^/48 where *l*_me_ is the thickness of the membrane with *l*_me_ ≃ 5d in all cases.

The dependence of the spontaneous curvature *m* on the solute concentration Φ_S_ is displayed in Figure 4b. For both good and poor solvent conditions, the spontaneous curvature *m* increases monotonically with the solute concentration. Comparing the *m* values for the two solvent conditions, we conclude that the spontaneous curvature increases more strongly for poor solvent conditions, as expected from the stronger increase of the solute coverage in Figure 3b. A simple estimate for this enhanced increase of the spontaneous curvature can be obtained from the straight fitting lines in Figure 4b. To do so, we consider the excess spontaneous curvature *Δm* ≡ *m* (Φ_S_) – *m* (Φ_S_ = 0) with the spontaneous curvature *m* (Φ_S_ = 0) = 0.089/*d* as obtained in the absence of solute; see Table S1. The excess quantity *Δm* behaves as *Δm* = *b*_m_Φ_S_/*d* with the dimensionless coefficient *b*_m_ = 1.08 for good and *b*_m_ = 6.04 for poor solvent conditions. Therefore, for the same solute concentrations, the excess spontaneous curvature *Δm* is about 5.6 times larger for poor solvent conditions with solubility ζ = 25/40 than for good solvent conditions with solubility ζ = 25/32.

### Recurrent Opening and Closure of Membrane Necks

**Figure 5:**
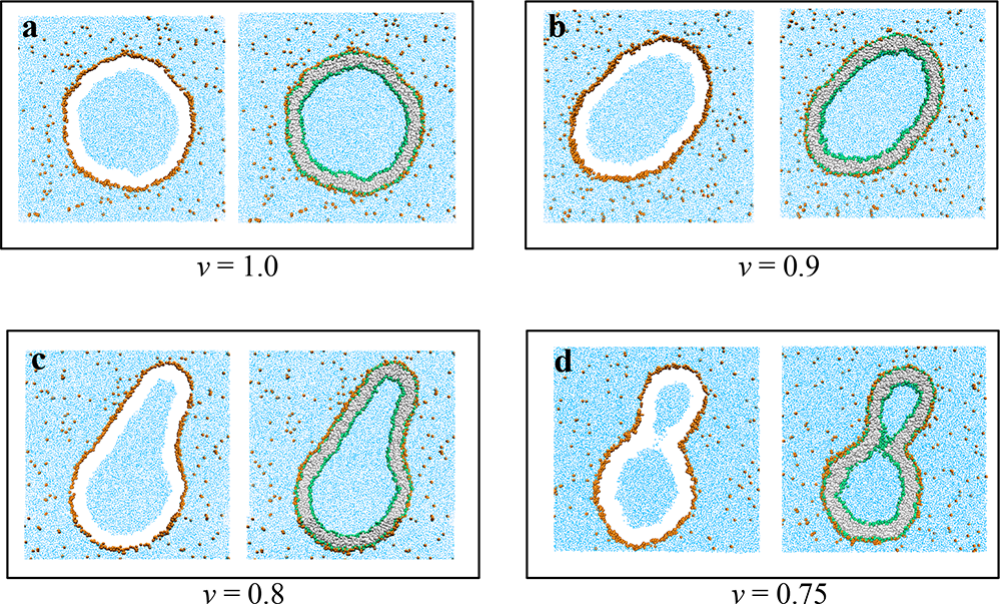
Deflation of nanovesicle exposed to exterior solution with solute concentration Φ_S_ = 0.025 close to the binodal. (a) Spherical vesicle with volume parameter ν = 1.0. (b) Prolate vesicle with ν = 0.9. (c) Pear-shaped vesicle with ν = 0.8. (d) Budded vesicle with ν = 0.75. For each ν value, two views of the same snapshot are presented: the views on the right represent the cross sections of the whole simulation box, and those on the left represent the same cross sections but with the lipids removed to improve the resolution of the solute monolayers (orange). The snapshots in (a)–(c) display stable equilibrium shapes for different ν values. In contrast, the membrane neck in (d) undergoes recurrent shape changes between open and closed states, as revealed by the time series in Figure 6.

**Figure 6:**
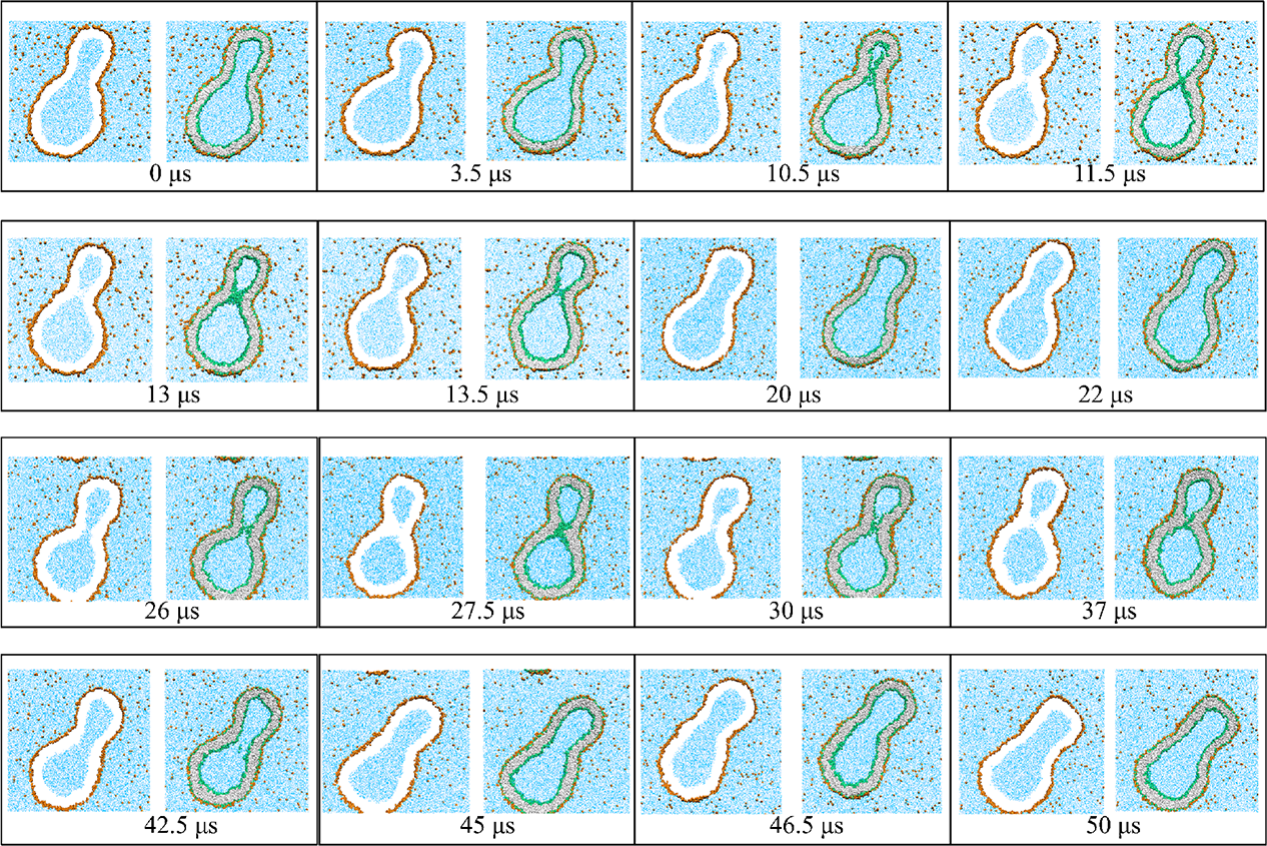
Time evolution of an individual nanovesicle exposed to solute concentration Φ_S_ = 0.025 close to the binodal. At *t* = 0 μs, the volume of the vesicle is reduced from ν = 0.80 to ν = 0.75 and then remains constant at this latter value. The vesicle responds to this volume decrease by closing and reopening its neck in a recurrent fashion, see also the time-lapse Movie 1, which shows these shape changes in more detail for the time interval between 1 μs ≤ *t* ≤ 47 μs. This recurrent process of neck closure and neck opening persists for at least 90 μs, as demonstrated in Figure 7, which displays the corresponding time evolution of the neck diameter.

**Figure 7:**
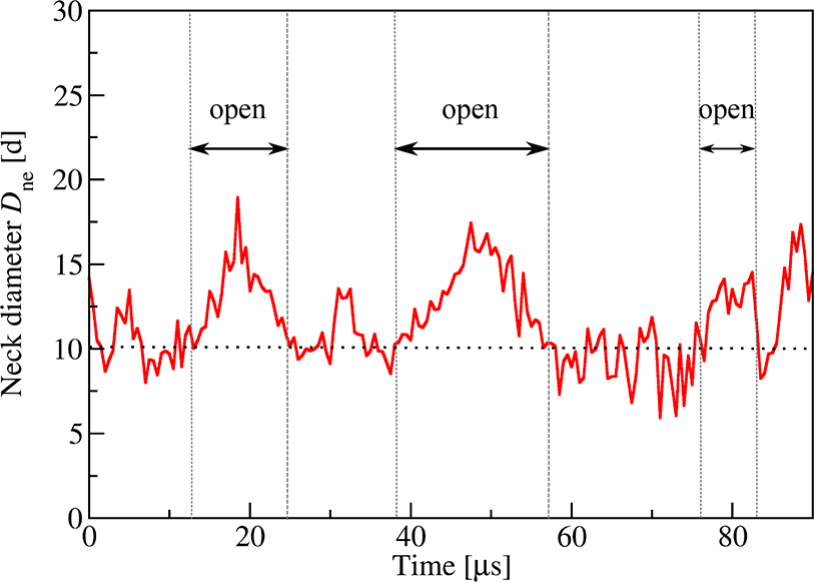
Time evolution of outer neck diameter *D*_ne_ corresponding to the snapshots of the nanovesicles in Figure 6 for volume ν = 0.75. The membrane neck repeatedly closes and opens up again. By definition, the neck is closed for *D*_ne_ < 10*d* and open for *D*_ne_ > 10*d*.

We now consider poor solvent conditions with solubility ζ = 25/40 and increase the solute concentration to mole fraction Φ_S_ = 0.025, which is close to the binodal line at Φ_S_ = 0.0275. In this case, the vesicle is observed to form a budded shape for ν = 0.75 as shown in Figure 5. However, for these parameters, the vesicle does not attain a stable shape but undergoes persistent shape changes with recurrent closure and opening of the membrane neck; see the time series of snapshots in Figure 6. The corresponding time series of the outer neck diameter is displayed in Figure 7. This recurrent behavior is reminiscent of the flickering fusion pores (kiss-and-run) that have been described for synaptic vesicles.^37−39^

Intuitively, one would expect that the unusual shape changes of the neck as displayed in Figures 6 and 7 arise from the vicinity of the binodal line. In order to corroborate this expectation, we performed control simulations to find out whether we observe similar shape changes of the neck for good solvent conditions as well. As an example, we studied the behavior of the membrane neck as we increased the solute concentration from Φ_S_ = 0.08 to Φ_S_ = 0.1, for constant vesicle volume ν = 0.7 and good solvent conditions as in Figure 2b. As shown in Figure S7, the neck closes after about 2 μs and then undergoes rather small fluctuations with the outer neck diameter *D*_ne_ confined to the interval 8*d* ≤ *D*_ne_ ≤ 10*d*.

### Budding, Membrane Adhesion, And Membrane Fission

**Figure 8:**
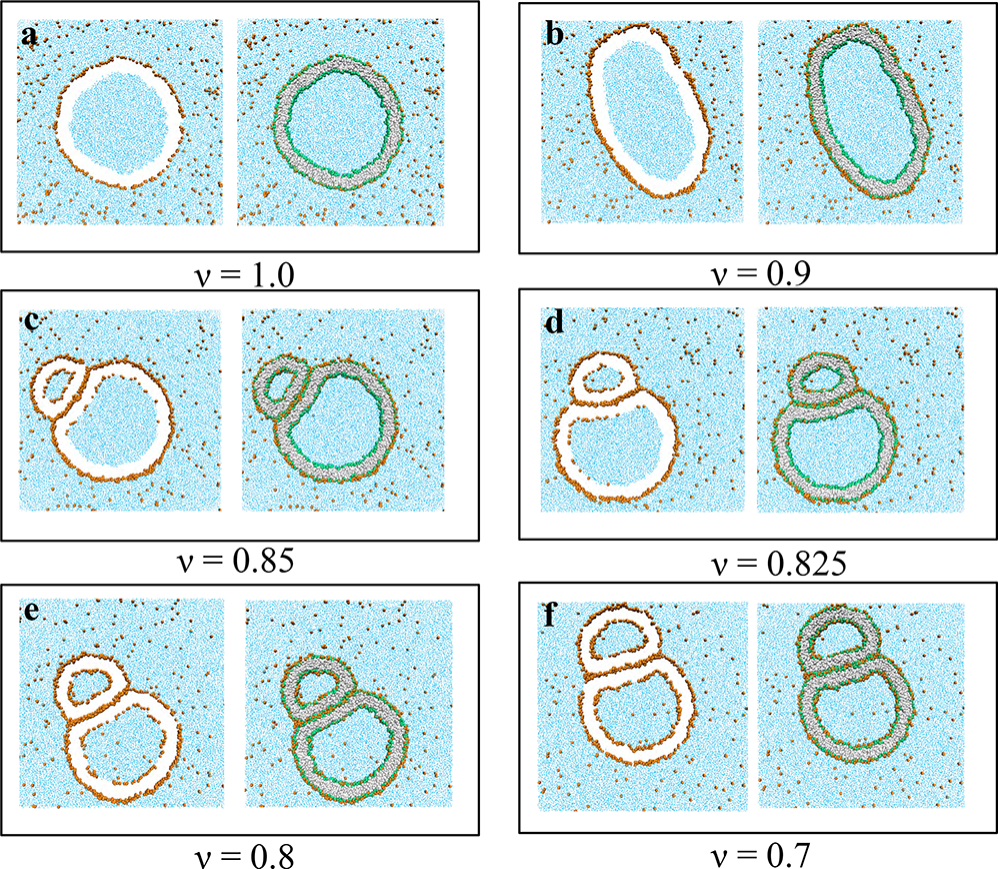
Stable shapes of an individual nanovesicle exposed to an exterior solution with solute concentration Φ_S_ = 0.026, poor solvent conditions. (a) Initial spherical vesicle with reduced volume ν = 1.0. (b) Prolate vesicle with ν = 0.9; (c–f) For ν = 0.85, 0.825, 0.8, and 0.7, two membrane segments close to the neck undergo adhesion, followed by neck fission, leading to two daughter vesicles that adhere *via* an intermediate layer of adsorbed solute (orange dots). For ν = 0.85, the corresponding time series of cross-sectional snapshots is displayed in Figure 9.

When we slightly increase the solute concentration to Φ_S_ = 0.026, which is very close to the binodal line, the nanovesicle undergoes a complex budding process with subsequent membrane adhesion and neck fission provided the volume is below the threshold value, ν = 0.85. This threshold for the volume can be deduced from the stable vesicle shapes as shown in Figure 8 for different ν values. For ν = 0.9, the vesicle attains a stable prolate shape. In contrast, for smaller volumes with ν ≤ 0.85, the vesicle divides into two daughter vesicles which adhere to one another by an intermediate adsorption layer of solute. This adhering couple of vesicles represents the stable vesicle morphology for the whole run time of the simulations, typically 100 μs.

**Figure 9:**
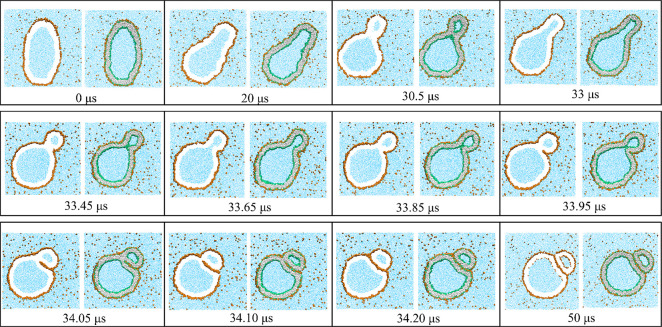
Nanovesicle exposed to exterior solution with solute concentration Φ_S_ = 0.026, poor solvent conditions: At time *t* = 0 μs, we start from a prolate shape with volume ν = 0.9 and reduce the vesicle volume to ν = 0.85, which is then kept fixed for all later times. The vesicle transforms into a dumbbell shape with a membrane neck that is closed at *t* = 30.5 μs and reopens fast within about one μs. The cross-sectional snapshot at *t* = 33.85 μs indicates that the geometry of the neck has changed by adhesion of two membrane segments close to the neck. This adhesion is mediated by a layer of adsorbed solutes (orange dots), which generate a rapidly expanding contact area until the neck is cleaved and the vesicle is divided into two daughter vesicles. These two separate vesicles continue to adhere to each other *via* an intermediate adsorption layer of solutes and form a stable morphology for later times *t* ≥ 34.20 μs. The time dependence of the neck diameter and the growing contact area are displayed in Figures 10 and 11, respectively.

One example for such an adhesion-induced division of the nanovesicle is displayed in Figure 9. This figure shows a time series of cross-sectional snapshots for volume ν = 0.85, starting from a prolate shape with volume ν = 0.9, which is deflated to ν = 0.85 at time *t* = 0 μs. We observe a neck closure event at time *t* = 30.5 μs and a subsequent reopening event within the next two microseconds, followed by the adhesion of two membrane segments close to the neck. The contact area between these two segments, which belong to the different subcompartments connected by the neck, grows rapidly in size until the membrane neck has been cleaved at *t* = 33.20 μs, leading to two separate nanovesicles that remain in contact with each other by the intermediate adsorption layer of solute.

**Figure 10:**
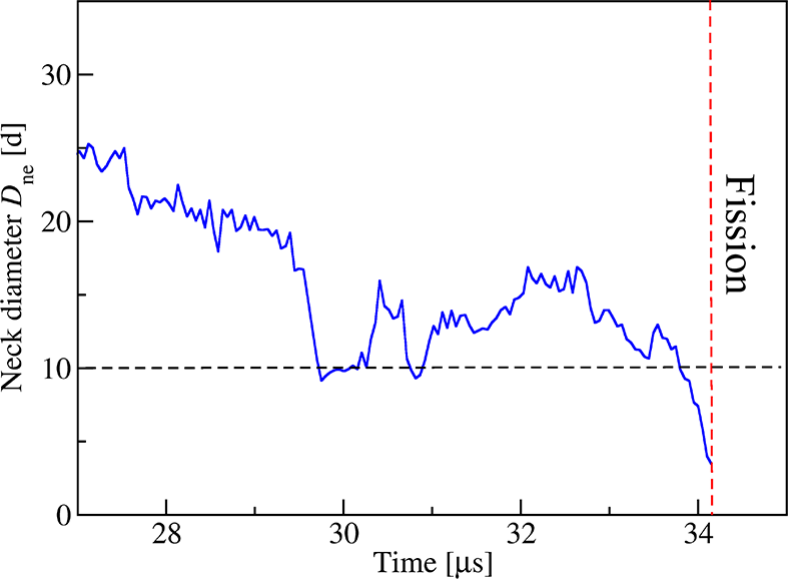
Time evolution of neck diameter *D*_ne_, corresponding to the time series of snapshots in Figure 9. In this example, the neck was cleaved and the nanovesicle divided after a fission time of 34.15 μs. Note that the fission process involves a free energy barrier that has to be overcome by thermal noise. Therefore, the fission time varies from one fission event to another; compare Figure S8.

**Figure 11:**
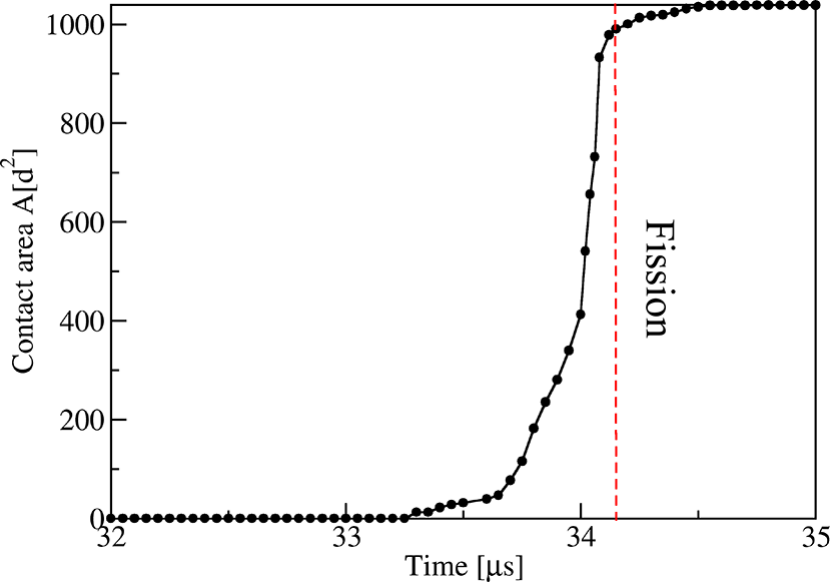
Time evolution of contact area between the two adhering membrane segments close to the neck: A small contact area has been formed at time *t* = 33.4 μs and starts to increase rapidly after *t* = 33.7 μs until the neck undergoes fission at *t* = 34.15 μs and the vesicle is divided up into two daughter vesicles that adhere to each other; see Figures 9 and 10. After division, the contact area rapidly attains the constant value 1039*d*^2^.

The process of neck fission and vesicle division is described quantitatively in Figures 10 and 11, which display the time evolution of the neck diameter and of the growing contact area, respectively. Inspection of Figure 10 shows that the neck diameter first undergoes thermally induced changes in close analogy to the behavior for solute concentration Φ_S_ = 0.025 in Figure 7. These shape changes of the neck involve two neck closure and reopening events in the time interval between *t* = 29 and *t* = 33 μs; see Figure 10. Furthermore, after *t* = 33.4 μs, the nanovesicle has developed a small contact area; see Figure 11. This contact area starts to grow rapidly after *t* = 33.7 μs and then becomes visible in the cross-sectional snapshots of Figure 9 and time-lapse Movie 2. Close inspection of this growing contact area reveals that it breaks the rotational symmetry of the dumbbell shape, which makes it more difficult to visualize its geometry.

Membrane fission has to overcome a free energy barrier arising from the local disruption of the lipid bilayer structure. The molecular details of this disruptive process are difficult to deduce from the simulation data. On the supermolecular scale, one may envisage the barrier for the fission of a membrane neck to be caused by the creation of two ring-like hydrophobic bilayer edges across the neck. Such a barrier has been previously discussed in the context of giant vesicles, for which the work needed to overcome this barrier can be expended by curvature-induced constriction forces.^5,40,41^ In contrast, for the fission of nanovesicles as considered here, this work is performed by the spreading of the solute-mediated contact area between the two membrane segments close to the neck. In both cases, the fission process is driven by thermal noise which implies that the fission time represents a random variable that varies between different fission events. To corroborate this stochastic aspect, we provide another example for neck fission in Figure S8 which is characterized by the fission time *t* = 28 μs.

### Comparison with Previous Simulation Studies on Fission

Fission of nanovesicles can be induced by several mechanisms as observed in a few previous simulation studies. When a nanoparticle is completely engulfed by the vesicle membrane, a neck is formed that may undergo fission as observed in Brownian dynamics simulations without explicit water.^42^ This process is governed by the adhesion between the membrane and the nanoparticle, which leads to an adhesion-dependent constriction force at the membrane neck.^43,44^ Fission has also been observed in coarse-grained molecular simulations of bilayers with two lipid components^45,46^ and in dissipative particle dynamics of monolayers assembled from triblock copolymers.^47^ The nanovesicle in ref (45) was formed by an asymmetric bilayer exposed to two different types of water beads in the interior and exterior compartment. The associated spontaneous curvature generates a constriction force that can be sufficiently large to cleave the neck^40,41^ as experimentally observed for giant vesicles.^5^ The latter mechanism also applies to the fission of one-component monolayers of asymmetric block copolymers.^47^ The two-component bilayers and monolayers in refs (46) and (47) consisted of two membrane domains with the domain boundary being located within the membrane neck of the dumbbell-shaped vesicles. In such a situation, the cleavage of the neck depends both on the line tension of the domain boundary^48^ and on the spontaneous curvatures of the two membrane domains. Furthermore, using coarse-grained molecular models, the cleavage of membrane necks by protein helices mimicking dynamin has been studied by simulations as well.^49^

In the present study, we introduced and explored a different fission mechanism based on the adsorption of small solutes. The spontaneous curvature generated by the solutes leads to vesicle budding and neck formation. In addition, close to the binodal line, the neck is cleaved by solute-mediated adhesion of the two membrane segments on opposite sides of the neck. Our quantitative analysis shows that this fission mechanism can be controlled by two key parameters, the solubility and the concentration of the solute in the exterior solution.

## Conclusions

In this paper, we studied the morphological responses of nanovesicles exposed to small solutes that adsorb onto the outer leaflet of the vesicle membranes. The vesicle shapes were shown to depend on three control parameters: the vesicle volume, the solute concentration in the exterior solution, and the solvent conditions. We distinguished good solvent conditions, for which the solution forms a uniform liquid phase for all solute concentrations, from poor solvent conditions, which lead to phase separation of the solution for a certain intermediate range of solute concentrations (Figure S1). For good solvent conditions, the nanovesicles formed prolate and dumbbell shapes with closed membrane necks. The formation of such closed necks was obtained by two different pathways (Figure 2) and was shown to be reversible (Figure S6). In particular, we showed that a dumbbell shape with a closed membrane neck can be attained from a prolate shape by simply increasing the solute concentration in the exterior solution.

For small solute concentrations, the morphological transformations of the nanovesicles are quite similar to those that have been observed in the absence of solute. Thus, in this concentration regime, the behavior of the nanovesicles is hardly affected by the solvent conditions (Figures S4 and S5). However, when we studied poor solvent conditions and solute concentrations close to the binodal line, at which point the solution starts to undergo aqueous phase separation, we observed very unusual behavior of the nanovesicles. As we approached this line from the uniform one-phase region of the aqueous solution, we first found a regime in which the nanovesicle undergoes recurrent shape changes between dumbbells with open and closed necks. One example was studied for solubility ζ = 25/40 and solute concentration Φ_S_ = 0.025, as displayed in Figures 6 and 7 as well as in the time-lapse Movie 1. As we approached the binodal line even closer for the same solvent conditions, we observed solute-induced fission of the closed neck and division of the nanovesicle into two daughter vesicles that adhere to each other by an intermediate adsorption layer of solute.

Such a fission and division process was observed for poor solvent conditions (ζ = 25/40) and solute concentration Φ_S_ = 0.026 whenever the volume parameter ν was comparable or smaller than 0.85 as shown in Figure 8. One example for the latter process is displayed in Figure 9 and time-lapse Movie 2, which correspond to volume parameter ν = 0.85. In this example, the fission started after about 34 μs as can be concluded from the time course of the neck diameter displayed in Figure 10. Inspection of this figure shows that the neck initially closes and reopens again, similar to the shape changes in Figure 7, but these closure and opening events are eventually truncated by neck fission. The underlying fission mechanism is provided by the solute-mediated adhesion of the two membrane segments close to the neck, which leads to a growing contact area between these two segments as shown in Figure 11.

Both the recurrent shape changes and the fission of the membrane neck can be understood when we envisage a certain dependence of the vesicle’s free energy landscape on the solute concentration. As we approach the binodal line, the free energy landscape develops local minima, which are relatively shallow, have a similar depth, and are separated by low free energy barriers that can be easily overcome by thermal noise. The simplest landscape, which leads to recurrent shape shapes of the neck as in Figures 6 and 7, has two such local minima, one minimum with an open and the other one with a closed neck. In the latter case, the shape changes of the nanovesicle involve both forward transitions from open to closed necks as well as backward transitions from closed to open necks (Figure 7).

For Φ_S_ = 0.026, on the other hand, the free energy landscape exhibits yet another local minimum corresponding to two daughter vesicles that adhere to each other by an intermediate adsorption layer of solute, as depicted in panels c–f of Figure 8. This additional local minimum can be reached from the dumbbell state with a closed neck *via* a topological transformation. The free energy barrier between the closed-neck dumbbell and the divided nanovesicle arises from the local disruption of the lipid bilayer structure. On the supramolecular scale, one may envisage this barrier to describe the work that is needed to create two ringlike hydrophobic bilayer edges across the neck membrane.^40,41^ One important objective for future studies is to explicitly calculate the free energy landscape for the fission process reported here by superimposing the contributions from membrane elasticity, which involves both membrane bending and stretching, as well as from the solute-mediated adhesion that induces the contact area of the self-adhering vesicle membrane. Preliminary simulation studies indicate that we may need more than one reaction coordinate in order to describe the essential features of this free energy landscape.

Fission of membrane necks can also be induced by other mechanisms. One such mechanism is the constriction force generated at the membrane neck by a sufficiently large spontaneous curvature.^5^ When the neck connects two membrane domains that differ in their lipid composition, fission is further facilitated by the line tension^48^ of the domain boundary. Furthermore, the fission of a neck that forms during endo- and exocytosis of nanoparticles experiences a constriction force that depends on the adhesive strength between the particle and the membrane.^43,44^ The latter mechanism explains membrane fission as observed by Brownian dynamics simulations in ref (42) and should also apply to the engulfment of active nanoparticles as studied in ref (50). If the nanoparticle is engulfed by an intramembrane domain (or raft), the fission is again promoted by the line tension of the domain boundary as observed in dissipative particle dynamics simulations.^51^

In the present study, we observed solute-induced budding and fission of nanovesicles using coarse-grained molecular dynamics simulations. The same processes should also be accessible to experiments on real nanovesicles. The first steps of such an experimental study would be to deflate the nanovesicles into prolate shapes and then to expose these shapes to an increasing solute concentration in the exterior solution. Using such a protocol, one should be able to obtain nanovesicles that form dumbbells with closed membrane necks. Based on our simulations, the formation of dumbbell shapes is predicted to be experimentally feasible for both good and poor solvent conditions. On the other hand, the solute-mediated fission of the dumbbell-shaped nanovesicles as observed in the simulations requires poor solvent conditions and solute concentrations close to the binodal line at which the aqueous solution undergoes phase separation.

## Methods

### Molecular Modeling and Dynamics

We study nanovesicles with a diameter of about 36 nm. These vesicles are exposed to small solute molecules in the exterior aqueous solution. The equilibration of these systems requires simulation times of many microseconds. To gain access to these length and time scales, we use a coarse-grained molecular model which we study by dissipative particle dynamics.^52^ Our molecular system is built up from four types of beads that represent small molecular groups corresponding to water (W) beads, lipid chain (C) beads, lipid head (H) beads, as well as solute (S) beads. The lipid molecules have a headgroup consisting of three H beads and two hydrocarbon chains, each of which consists of six C beads. All beads have the diameter *d*, corresponding to about 0.8 nm. Each pair of beads interacts with short-ranged pairwise additive forces as described previously,^52^ including the conservative force
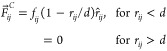
6with the force parameter *f*_*ij*_, the unit vector *r̂*_*ij*_ pointing from bead *j* to bead *i*, and the distance *r*_*ij*_ between bead *i* and bead *j*. The numerical values of the force parameters *f*_*ij*_ are displayed in Table 1. If we delete the right-most column with force parameters *f*_*iS*_ and the bottom row with *f*_*Sj*_, we recover the force parameters that have been used before^19^ in the absence of solute.

**Table 1:** DPD Force Parameters *f*_*ij*_ in Units of *k*_B_T/*d*^a^

*f*_*ij*_	H	C	W	S
H	30	50	30	15
C	50	10	75	75
W	30	75	25	*f*_WS_
S	15	75	*f*_SW_	25

a The system is built up from lipid head (H) beads, lipid chain (C) beads, water (W) beads, and solute (S) beads. The values for *f*_HS_ = *f*_SH_ and *f*_WS_ = *f*_SW_ have been changed compared to ref (52) in order to increase the affinity of the solutes to the lipid head groups. The force parameter *f*_WS_ = *f*_SW_ is taken to be 32 and 40 for good and poor solvent conditions, respectively.

In the computational approach used here, the solubility can be measured by the force parameter ratio
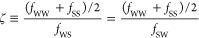
7which involves the two force parameters *f*_WW_ and *f*_SS_ between a pair of water and solute beads as well as the force parameter *f*_WS_ = *f*_SW_ for a water bead interacting with a solute bead; compare Table 1. As we increase *f*_WS_, contacts between water and solute beads become energetically less favorable. These contacts can be avoided by the segregation of the two types of beads. As a consequence, decreasing the solubility ζ by increasing *f*_WS_ can lead to phase separation into a solute-poor and a solute-rich phase.

In order to perform the simulations of nanovesicles in aqueous solution, we have used LAMMPS (large scale atomic/molecular massively parallel simulator)^53^ which is an efficient and parallelized classical molecular dynamics simulator. We study a cuboid simulation box, each side of which has a linear dimension of 80*d*, leading to the box volume (80*d*)^3^. The total number of beads inside the box is 1591900. The bulk water density is kept fixed at ρ = 3/*d*^3^, which ensures the bulk pressure to be *P* = 20.7*k*_B_*T*/*d*^3^ arising solely from the contribution of the molecular interactions, i.e., without the constant contribution of 3 *k*_B_*T*/*d*^3^ arising from the kinetic energy.

Our simulations started from spherical nanovesicles which were assembled by placing *N*_il_ lipids onto the inner leaflet and *N*_ol_ lipids onto the outer leaflet of the bilayer. All simulations described in this paper were performed for *N*_il_ = 4 000 and *N*_ol_ = 6 100, corresponding to the lipid asymmetry parameter . Each lipid molecule was built up from 3 H and 12 C beads, which implies that the lipid bilayers consisted of 151500 H and C beads. Therefore, the interior and exterior aqueous solution contained 1440400 W and S beads, with all S beads located in the exterior solution.

In order to calculate the equilibrium properties of the nanovesicle, we initially equilibrated the system using the NPT ensemble for about 2 μs, thereby adjusting the total bulk pressure to the value *P* = 23.7*k*_B_T/*d*^3^. Subsequently, we switched to the NVT ensemble and performed the final equilibration for a typical run time of 10 μs. We discarded the first 5 μs of the DPD trajectory and used the remaining 5 μs to calculate the equilibrium properties (stress profile, density profile, solute coverage Γ, and spontaneous curvature *m*) by averaging over 10 bins of length 0.5 μs. In order to observe the shape transformations of the nanovesicles induced by changes in vesicle volume, we had to perform much longer simulations with run times up to 50 μs (Figures 6 and 9) or even 90 μs (Figure 7)

### Solute Mole Fraction and Solute Density

The solute mole fraction Φ_S_ represents a global quantity that can be directly controlled during the initial setup of the system and remains constant because the lipid bilayer is essentially impermeable to both water and solute beads on the time scale of the simulations. We will also use the solute density ρ_S_ to characterize the local solute concentration which will, in general, depend on both space and time. For the special case of a time-independent, spatially uniform solution with volume *V*, the solute density ρ_*S*_ ≡ *N*_S_/*V* whereas the total density ρ = (*N*_S_ + *N*_W_)/*V* which implies the relationship ρ_S_ = Φ_S_ ρ. In all our simulations, we used the standard value ρ = 3/*d*^3^ for the total density,^52,54^ which implies the relationship ρ_S_ = 3Φ_S_/*d*^3^ between the solute density ρ_S_ and the solute mole fraction Φ_S_ for a uniform exterior solution.

### Pressure Tensor and Stress Profile of Spherical Nanovesicle

Because of the spherical symmetry, the local stress or pressure tensor has the general form^19^
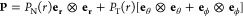
8with the normal component *P*_N_(*r*) and the tangential component *P*_T_(*r*) where **e**_*r*_, **e**_θ_, and **e**_ϕ_ are orthogonal unit vectors and the symbol ⊗ represents the dyadic product. The numerical values of *P*_N_(*r*) and *P*_T_ (*r*) as well as the stress profile
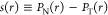
9can be calculated using the computational method described in refs (55) and (56). The bilayer tension Σ can then be obtained by spatial integration of the stress profile, leading to^19^
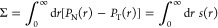
10in close analogy to the interfacial tension^57^ of a spherical liquid droplet.

### Relaxed States of Nanovesicles with Tensionless Bilayers

Fluid membranes can be elastically deformed by stretching and bending. To calculate the corresponding elastic moduli—the area compressibility modulus *K*_A_, the bending rigidity κ, and the spontaneous curvature *m*—we need to determine the elastically relaxed state of the vesicle, in which the bilayer tension as given by eq 10 is close to zero.

In order to obtain such a relaxed state of the nanovesicle, we started from initially assembled vesicles with *N*_W_^isp^ = 90 400 enclosed water beads and varied the latter bead number and thus the vesicle volume for fixed lipid numbers *N*_il_ and *N*_ol_ in the two bilayer leaflets as well as for fixed solute concentration in the exterior solution. From the variation of the bilayer tension with vesicle volume, we can determine the number of interior water beads, *N*_W_^in^ = *N*_W0_^in^, for which the vesicle membrane has (almost) zero bilayer tension. The corresponding volume parameters, ν = ν_0_ ≡ *N*_W0_^in^/*N*_W_^isp^, are displayed in Tables S1 and S2 for good and poor solvent conditions, respectively.

### Elastic Properties of Bilayer Membranes

In order to determine the stress asymmetry or spontaneous curvature *m* of a spherical vesicle membrane from eq 5, we need to calculate the radius *R*_mid_ of the vesicle’s midsurface. As explained in ref (19), we have several options to define this midsurface. The simplest option is to locate the midsurface at the peak of the density profile ρ_C_(*r*) for the hydrocarbon chains which are represented here by chain (C) beads. The resulting midsurface radius *R*_mid_ is displayed in Tables S1 and S2 for good and poor solvent conditions as well as vanishing bilayer tension. The area *A*_0_ of the tensionless bilayer is then calculated from *A*_0_ = 4*πR*_mid_^2^, and the area compressibility modulus *K*_A_ of the bilayer is obtained from the asymptotic equality
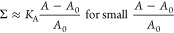
11This area compressibility modulus is found to decrease with increasing solute mole fraction Φ_S_, see the seventh columns of Tables S1 and S2, which implies that the adsorbed solutes facilitate membrane stretching and soften the membrane.

We calculate the bending rigidity κ of the membrane using the relationship^58^
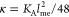
12with the bilayer thickness l_me_ ≃ 5*d*, which was found to apply to all solute concentrations. The numerical prefactor 1/48 in eq 12 has been controversial for some time^59,60^ but has been recently confirmed^36^ by the fluctuation spectra of two-component bilayers for a wide range of lipid compositions. The bending rigidity κ as obtained from the relationship in eq 12 also decreases as a function of increasing solute concentration Φ_S_^ex^, see eighth columns of Tables S1 and S2, reflecting the decrease of the area compressibility modulus.

## Supporting Information Available

Supporting Figures S1–S8, which display the phase diagram for the water-solute binary liquid mixture; the time evolution of solute-rich droplets in the one-phase and two-phase region of the mixture; shape transformations for low solute concentrations; reversible neck closure and opening of a budded nanovesicle; small fluctuations of neck diameter for good solvent conditions; and another example for neck fission; Supporting Tables S1 and S2 with concentration-dependent properties of nanovesicles for good and poor solvent conditions (PDF)
Recurrent shape changes of dumbbell-like shape (AVI)
Neck fission of dumbbell-like shape (AVI)

## Supplementary Material

nn1c00525_si_001.pdf

nn1c00525_si_002.avi

nn1c00525_si_003.avi
