# Supplementary material for: Budding and Fission of Nanovesicles Induced by Membrane Adsorption of Small Solutes
Source: ACS Nano. 2021 Apr 5;15(4):7237–48. doi: 10.1021/acsnano.1c00525 (PMC8155335; doi:10.1021/acsnano.1c00525)
Supplement: Supplementary file 1 — nn1c00525_si_001.pdf [file nn1c00525_si_001.pdf]

Supporting Information:

Budding and Fission of Nanovesicles Induced by Membrane Adsorption of Small Solutes

Rikhia Ghosh, Vahid Satarifard, Andrea Grafmüller, and Reinhard Lipowsky*

Theory & Biosystems, Max Planck Institute of Colloids and Interfaces, 14424 Potsdam, Germany

March 31, 2021

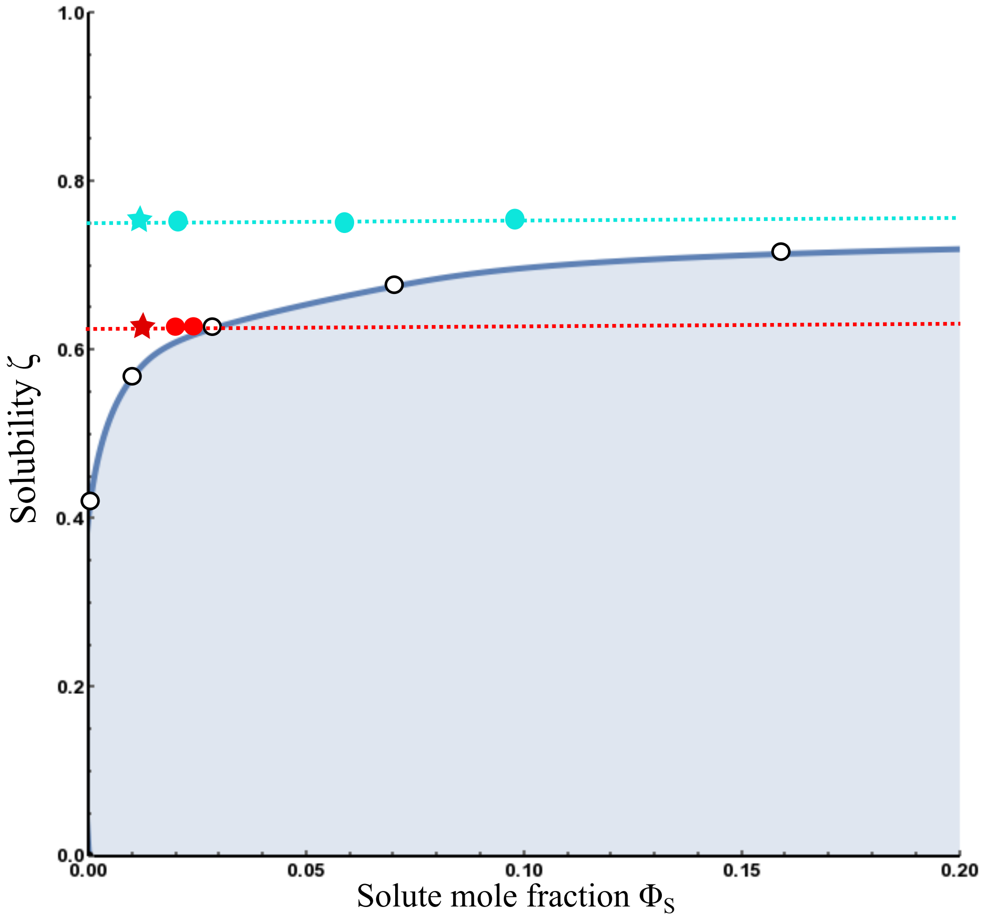

Figure S1: Phase diagram for the water-solute binary mixture as a function of solute mole fraction Φ_S and solubility ζ, see equs 2 and 7 in the main text. The open circles ($\bigcirc$, black) represent the data points for which the location of the binodal line has been explicitly determined. The solid line (dark blue) corresponds to the binodal as obtained by interpolation of the open circles. This binodal line separates the one-phase (white) from the two-phase region (greyish blue). The horizontal dotted line (light blue) corresponds to good solvent condition with $\zeta = \frac{25}{32} \simeq 0.781$, the horizontal dashed line (red) to poor solvent conditions with $\zeta = \frac{25}{40} \simeq 0.625$. The two stars ($\star$, blue and red) represents solutions with $\Phi_S = 0.016$, a small solute concentration for which the vesicle morphologies become essentially independent of solubility. The solid circles ($\bullet$, blue and red) represent solutions for which we have studied the vesicle morphologies as a function of the volume parameter ν as defined by eq 1 in the main text.

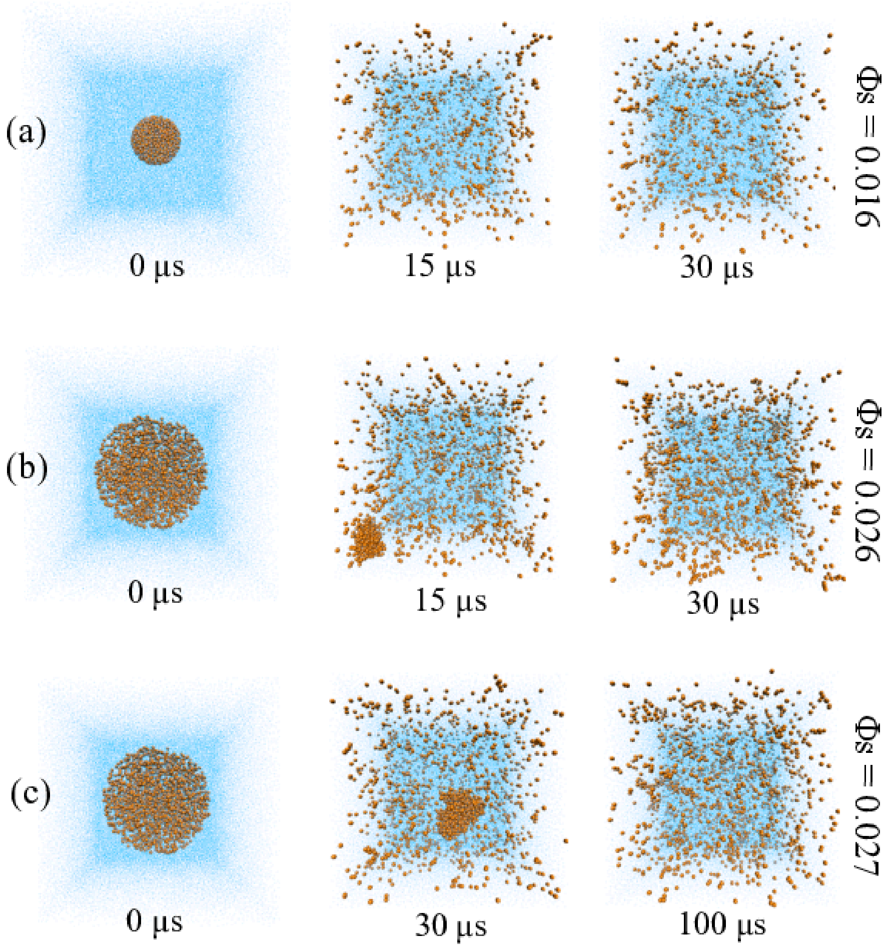

Figure S2: Time evolution of solute-rich sphere (orange) for poor solvent conditions ($\zeta = 25/40$) and three different mole fractions Φ_S that belong to the one-phase region of the water-solute binary mixture. For each Φ_S-value, the leftmost snapshot shows the initial sphere (orange) of solute beads at time $t = 0\mu s$ followed by two snapshots at later times. (a) For mole fraction $\Phi_S = 0.016$, the solute droplet has an initial radius of $4d$ and dissolves within $15\,\mu$s; (b) For $\Phi_S = 0.026$, the solute-rich droplet, which has an initial radius of $8d$, is still clearly visible after $15\,\mu$s but has dissolved after $30\,\mu$s; and (c) For $\Phi_S = 0.027$, the solute droplet with an initial radius of $8d$ shrinks during the first 30μs and has completely dissolved after 100μs. For visual clarity, the S beads are displayed as orange 'particles' and the W beads as blue 'points' even though both types of beads have the same diameter d.

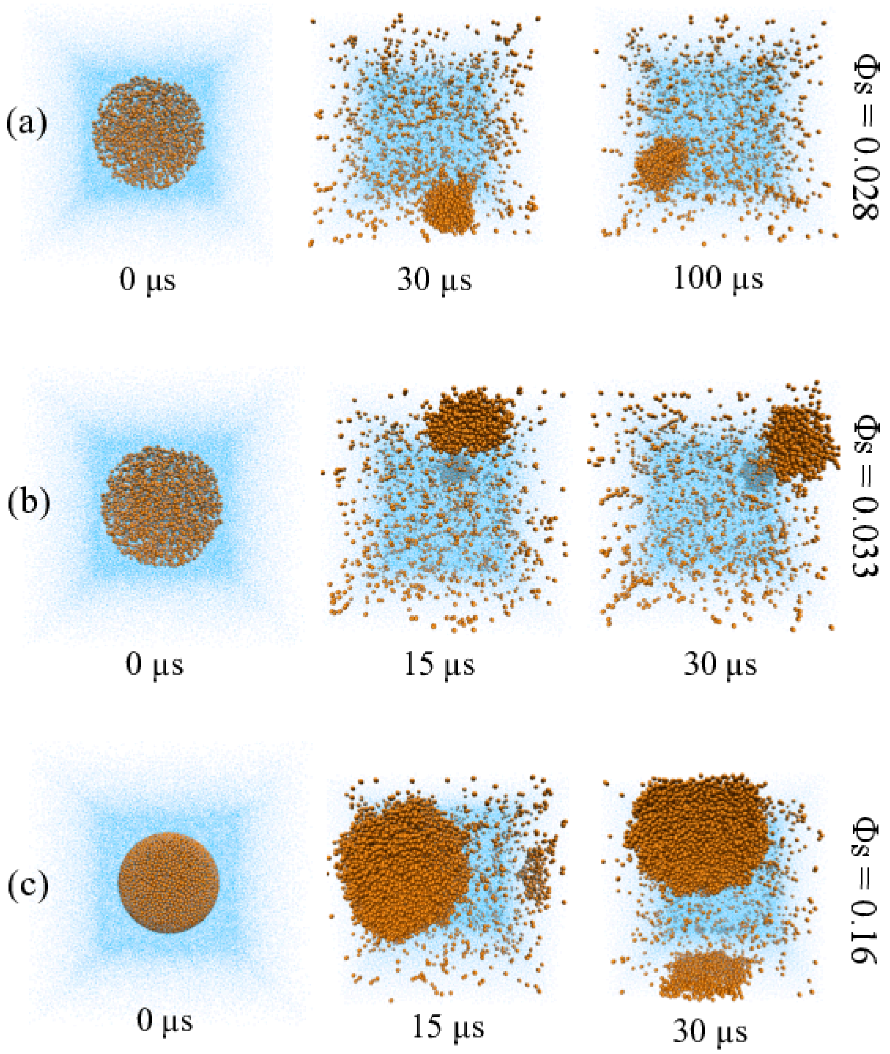

Figure S3: Time evolution of solute-rich sphere (orange) for poor solvent conditions ($\zeta = 25/40$) and three different mole fractions Φ_S that belong to the two-phase region of the water-solute binary mixture. For each Φ_S-value, the left-most snapshot shows the initial sphere (orange) of solute with radius $8d$, followed by two snapshots at later times: (a) For mole fraction $\Phi_\mathrm{S} = 0.028$, the droplet volume shrinks strongly during the first 30μs but remains constant for time $t > 30\mu s$; (b) For $\Phi_\mathrm{S} = 0.033$, the droplet volume shrinks during the first $15\mu s$ and remains constant for $t > 15\mu$s; and (c) For $\Phi_\mathrm{S} = 0.16$, the droplet volume grows during the first $15\mu s$ and stays constant for $t > 15\mu$s.

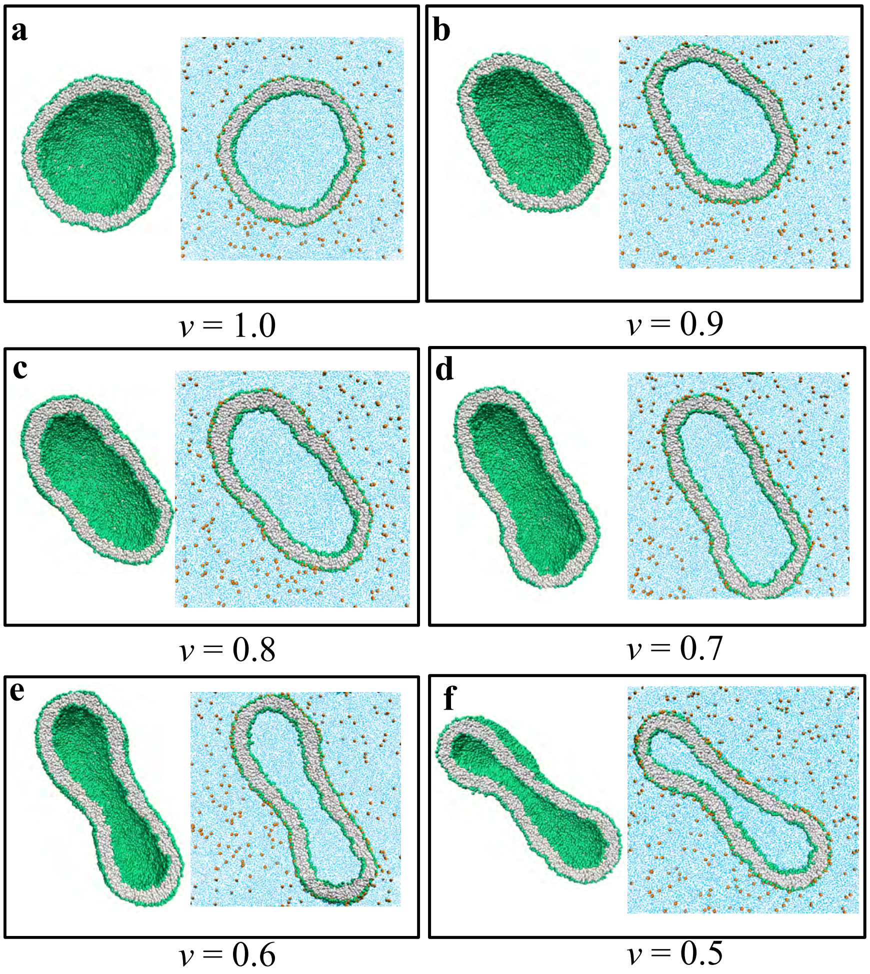

Figure S4: Vesicle morphologies exposed to an exterior solution with good solvent conditions ($\zeta = \frac{25}{32} \simeq 0.7352$) and solute mole fraction $\Phi_S = 0.016$: The initial sphere with $\nu = 1.0$ is transformed into a prolate upon deflation which becomes more and more elongated and evolves into a dumbbell-like shape as the volume is further reduced. Two views are displayed for each ν-value: on the left, half-views depicting only half of the vesicle membranes and, on the right, cross-sections of the whole simulation box.

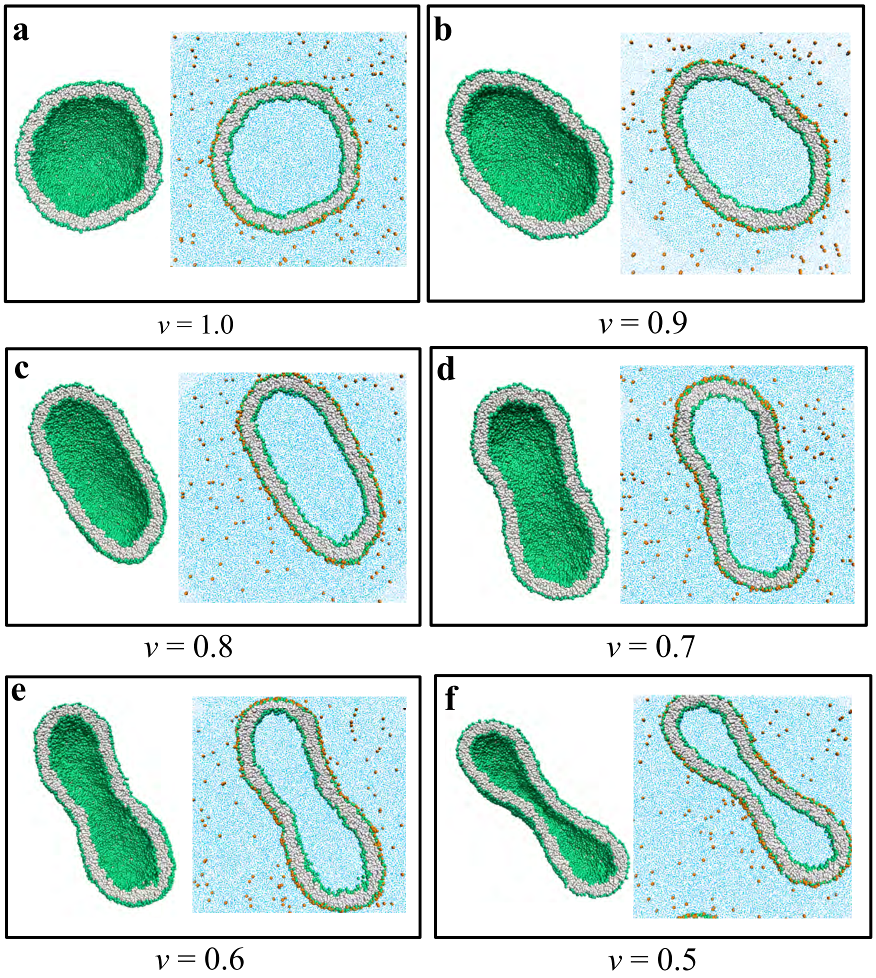

Figure S5: Shape transformation of nanovesicle exposed to an exterior solution with poor solvent conditions ($\zeta = \frac{25}{40}$) and solute mole fraction $\Phi_S = 0.016$: (a) Spherical vesicle with volume parameter $\nu = 1.0$; (b,c): Prolate vesicles with $\nu = 0.9, 0.8$; and (d,e,f) Dumbbell vesicles with $0.7, 0.6, 0.5$. For each ν-value, two views are displayed as in Figure S4.

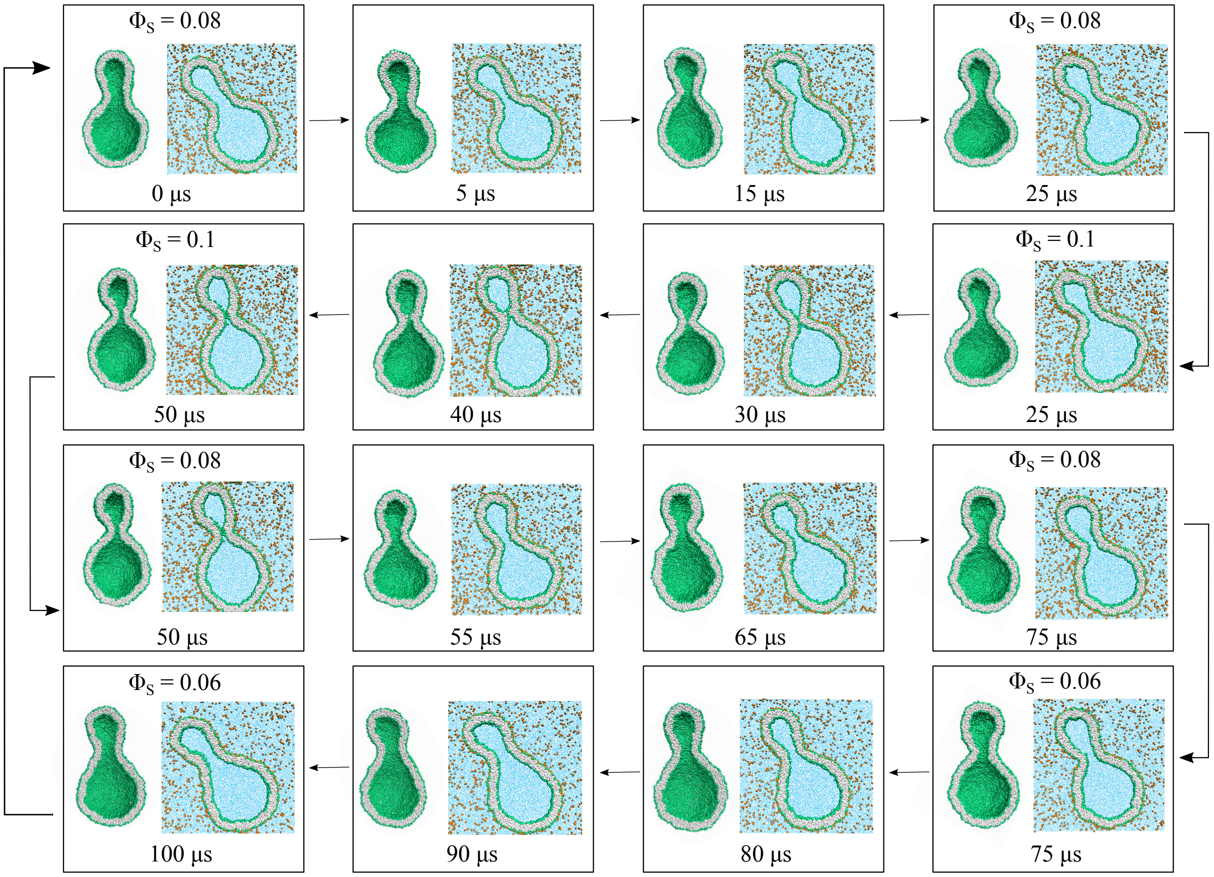

Figure S6: Reversible neck closure and opening of a budded nanovesicle with volume $\nu = 0.7$ for good solvent conditions. Initially, we start with a vesicle that was equilibrated for solute concentration $\Phi_S = 0.06$ but is exposed to the increased concentration $\Phi_S = 0.08$ at time $t = 0\,\mu$s. During the next 25 μs, the vesicle shape relaxes and reaches its new equilibrium state, corresponding to $\Phi_S = 0.08$. At $t = 25\,\mu$s, we further increase the exterior solute concentration from $\Phi_S = 0.08$ to $\Phi_S = 0.1$ and monitor the equilibration towards another equilibrium shape, corresponding to $\Phi_S = 0.1$. The latter shape is reached at $t = 50\,\mu$s and has a closed membrane neck. We now reverse the process by reducing the solute concentration to $\Phi_S = 0.08$ at $t = 50\,\mu$s, see left-most snapshot of the third row, and to $\Phi_S = 0.06$ at $t = 75\,\mu$s, see right-most snapshot of the last row. During these latter steps, the membrane neck opens up again until we reach the equilibrium shape for $\Phi_S = 0.06$ at $t = 100\,\mu$s. If we now increase the solute concentration again from $\Phi_S = 0.06$ to $\Phi_S = 0.08$, we reproduce the initial vesicle conformation at $t = 0\,\mu$s. Therefore, we can cycle through the whole neck closure and opening process again and again.

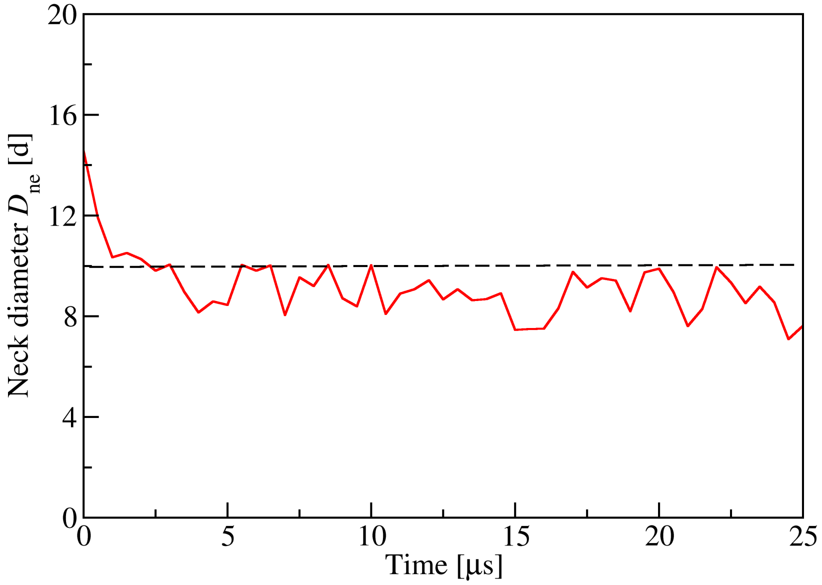

Figure S7: Time-dependent fluctuations of outer neck diameter D_{ne} after increasing the solute concentration from $\Phi_{\mathrm{S}} = 0.08$ to $\Phi_{\mathrm{S}} = 0.1$ for constant vesicle volume $\nu = 0.7$ and good solvent conditions ($\zeta = 25/32$). The neck closes after about $2\,\mu$s and then exhibits rather small fluctuations of its diameter D_{ne} within the range $8d \leq D_{\mathrm{ne}} \leq 10d$. This behavior is qualitatively different from the shape changes for poor solvent condition close to the binodal line, for which we observe recurrent neck closure and reopening events (Figure 7).

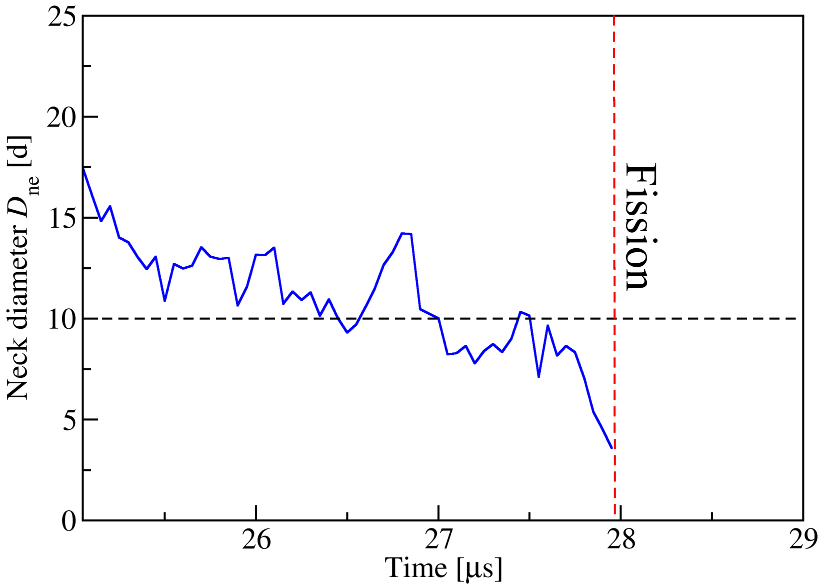

Figure S8: Another example for the fission of a membrane neck that connects two subcompartments of a nanovesicle exposed to solute concentration $\Phi_{\mathrm{S}} = 0.026$ and poor solubility ($\zeta = 25/40$), the same conditions as for the fission process in Figure 10. In the present example, the fission time is about $28\,\mu$s which differs from the fission time $34.15\,\mu$s observed in Figure 10, illustrating the stochastic nature of the process which involves a free energy barrier that has to be overcome by thermal noise.

Table S1: Spherical nanovesicles exposed to different solute concentrations Φ_S for *good* solubility $\zeta = 25/32$. All vesicle membranes contain $N_{il} = 4000$ lipids in the inner leaflet and $N_{ol} = 6100$ lipids in the outer leaflet. Tensionless bilayers are obtained for N_{W0}^{in} water beads enclosed by the vesicles. The corresponding volume parameters $\nu_0 \equiv N_{W0}^{in}/N_W^{isp}$ and bilayer tensions Σ are displayed in the third and fourth column. The radius R_{mid} of the midsurface is obtained from the peak of chain bead density. The coverage Γ is calculated by eq 4, the area compressibility modulus K_A by eq 12. The bending rigidity is computed using $\kappa = K_A l_{me}^2/48$ where l_{me} is the thickness of the membrane with $l_{me} \simeq 5d$ in all cases. The first moment of the stress profile, $\mathcal{T}_0$, and the spontaneous curvature m are obtained from eq 5. The data for $\Phi_S = 0$ are identical with those obtained by Ghosh *et al*, *Nano Lett.* **2019**, *19*, 7703-7711

Φ_S	N_{W0}^{in}	ν_0	$\Sigma\,[k_BT/d^2]$	$R_{mid}\,[d]$	$\Gamma\,[1/d^2]$	$K_A\,[k_BT/d^2]$	$\kappa\,[k_BT]$	$\mathcal{T}_0\,[k_BT/d]$	$m\,[1/d]$
0	87400	0.967	$+0.005 \pm 0.04$	22.02 ± 0.13	0	30	15	-1.35 ± 0.82	0.089 ± 0.030
0.008	87400	0.967	$+0.011 \pm 0.009$	22.02 ± 0.27	0.43 ± 0.02	28.14 ± 0.39	14.66 ± 0.58	-1.40 ± 0.41	0.093 ± 0.021
0.016	87400	0.967	-0.019 ± 0.015	22.15 ± 0.21	0.73 ± 0.06	24.49 ± 0.56	12.38 ± 0.23	-1.59 ± 0.35	0.110 ± 0.029
0.025	87900	0.972	-0.004 ± 0.009	22.25 ± 0.62	0.98 ± 0.05	23.78 ± 0.74	12.05 ± 0.41	-1.72 ± 0.21	0.116 ± 0.025
0.05	90400	1.0	-0.012 ± 0.026	22.40 ± 0.58	1.25 ± 0.05	23.14 ± 0.55	11.76 ± 0.20	-2.46 ± 0.51	0.149 ± 0.044
0.07	91300	1.009	$+0.017 \pm 0.011$	22.40 ± 0.91	1.35 ± 0.06	22.58 ± 0.27	9.73 ± 0.18	-3.51 ± 0.25	0.225 ± 0.028
0.09	92400	1.002	-0.006 ± 0.017	22.64 ± 0.29	1.61 ± 0.11	18.70 ± 0.45	9.28 ± 0.22	-3.88 ± 0.38	0.253 ± 0.031
0.1	93300	1.032	-0.011 ± 0.019	22.64 ± 0.63	1.76 ± 0.08	17.04 ± 0.58	8.88 ± 0.14	-4.13 ± 0.45	0.277 ± 0.034

Table S2: Spherical nanovesicles exposed to different solute concentrations Φ_S for *poor* solubility $\zeta = 25/40$. The different columns display the same quantities as in Table S1.

Φ_S	N_{W0}^{in}	ν_0	$\Sigma\,[k_BT/d^2]$	$R_{mid}\,[d]$	$\Gamma\,[1/d^2]$	$K_A\,[k_BT/d^2]$	$\kappa\,[k_BT]$	$\mathcal{T}_0\,[k_BT/d]$	$m\,[1/d]$
0.008	87400	0.967	$+0.021 \pm 0.019$	22.25 ± 0.16	1.04 ± 0.02	18.83 ± 0.66	9.80 ± 0.49	-1.59 ± 0.24	0.127 ± 0.021
0.016	88900	0.983	-0.009 ± 0.02	22.25 ± 0.31	1.99 ± 0.03	18.66 ± 0.29	9.71 ± 0.22	-2.02 ± 0.42	0.189 ± 0.029
0.025	90400	1.0	-0.019 ± 0.016	22.35 ± 0.11	3.01 ± 0.03	17.10 ± 0.52	8.91 ± 0.48	-2.67 ± 0.45	0.240 ± 0.043
0.026	90900	1.005	$+0.017 \pm 0.02$	22.40 ± 0.28	3.11 ± 0.05	14.10 ± 0.22	7.34 ± 0.17	-3.19 ± 0.29	0.262 ± 0.035

Supporting Movie Captions

Supporting Movie 1:

Time-lapse movie of budded nanovesicle for solute concentration $\Phi_S = 0.025$ and poor solvent conditions, see red line ($\zeta = 25/40$) in the phase diagram of Figure S1. Each snapshot of the movie represents a cross-section through the vesicle at time t as shown in the upper left corner. The membrane neck of the nanovesicle closes repeatedly (snapshots at times $t = 2\,\mu$s, $7\,\mu$s, $13\,\mu$, $26\,\mu$s, $30\,\mu$s, $35\,\mu$s, $37\,\mu$s, and $42\,\mu$s) and reopens again between these closure events. The movie displays these recurrent shape changes for the time interval between time $t = 1\,\mu$s and $t = 47\,\mu$s. A time series containing additional snapshots of the same nanovesicle is shown in Figure 6, see also Figure 7 with the corresponding time evolution of the membrane neck for the extended time interval $0 \leq t \leq 90\,\mu$s

Supporting Movie 2:

Time-lapse movie of budded nanovesicle for solute concentration $\Phi_S = 0.026$ and the same poor solvent conditions ($\zeta = 25/40$) as in Movie 1, see phase diagram of Figure S1. Each snapshot of the movie represents a cross-section through the vesicle at time t as displayed in the upper left corner. For this solute concentration, the membrane neck first undergoes a few closure and subsequent reopening events between $t = 29.0\,\mu$s and $t = 33.8\,\mu$s, qualitatively similar to the recurrent shape changes in Movie 1, but the last closure event at $t = 33.8\,\mu$s then leads to fission of the neck and division of the nanovesicle into two daughter vesicles that adhere to each other by an intermediate layer of adsorbed solutes, see the last couple of snapshots between $t = 33.8\,\mu$s and $t = 34.2\,\mu$s. A time series containing additional snapshots of the same nanovesicle is shown in Figure 9; the time evolution of the membrane neck diameter and the solute-mediated contact area is displayed in Figures 10 and 11, respectively.
